# On the Effect of Layering Velostat on Force Sensing for Hands

**DOI:** 10.3390/s25103245

**Published:** 2025-05-21

**Authors:** Tyler Bartunek, Ann Majewicz Fey, Edoardo Battaglia

**Affiliations:** 1 Department of Mechanical Engineering, The University of Utah, Salt Lake City, UT 84112, USA; 2 Department of Mechanical Engineering, The University of Texas at Austin, Austin, TX 78712, USA

**Keywords:** tactile sensing, Velostat, low-cost sensing, flexible sensors

## Abstract

Force sensing on hands can provide an understanding of interaction forces during manipulation, with applications in different fields, including robotics and medicine. While several approaches to accomplish this have been proposed, they often require relatively complex and/or expensive fabrication techniques and materials. On the other hand, less complex and expensive approaches often suffer from poor accuracy of measurements. An example of this is provided by sensors built with Velostat, a polyethylene–carbon composite material that exhibits resistance changes when force is applied. This material is both cheap and easy to work with, but sensors made from Velostat have been shown to suffer from low accuracy, limiting its usefulness. This work explores the effect of stacking multiple layers of 0.1 mm Velostat sheets on accuracy, using no additional fabrication techniques or other material aside from electrode connections, with the rationale that this is both economical and can be accomplished easily. We evaluate measurement error for designs with different numbers of layers (1, 3, 4, 5, 10, 20, and 30) against a load cell, and also compare this with the error for a USD 10 commercial force sensing resistor designed for measurement of hand forces (FSR 402) in three evaluations (static, cyclic, and finger base interactions). Our results show that layered sensors outperform both the one-layer design and the commercial FSR sensor consistently under all conditions considered, with the best performing sensors reducing measurement errors by at least 27% and as much as 60% when compared against the one-layer design.

## 1. Introduction

Tactile perception in people relies on sensory information gathered by mechanoreceptors embedded in the skin. It is most accurate in the hands, particularly on the fingertips and, to a lesser degree, the palm [1,2]. These perceptual capabilities enable us to use our hands to collect information that we use for object identification [3], exploration [4,5], and grasp state estimation while manipulating objects [1,2,6,7,8]. It is then not surprising that creating wearable systems that can quantify the forces involved during these tasks has received strong interest, and is used for a variety of applications, including the design and control of robots [3,6], medical applications [9,10,11,12] and others [10,11,13]. Within this context, the work presented in this paper was motivated by our work within a larger framework to establish touch-based bilateral remote communication between an expert surgeon and a trainee through wearable sensing and haptics [12,14,15] (a scenario which we will refer to as telementoring), for which we had the need to design custom flexible sensors for use with hands.

A recent review on wearable tactile sensors [9] summarized the most commonly used approaches based on their operating principles, which can be capacitive [16,17], piezoelectric [18], triboelectric [19], piezoresistive [3,20], optical [21], or hybrid [22]. Capacitive sensors typically are built with two plates separated by a dielectric, where differences in the capacitance measurements are primarily from deformations of the dielectric and resulting changes to the distance between the plates [16], or changes to the properties of the dielectric [17,23]. They have low power consumption and decent sensitivity but can be sensitive to external electromagnetic interference [9], exhibit creep behavior, require a preload, and generally need a relatively complex fabrication process and setup [24]. Piezoelectric tactile sensors leverage electric fields that are created from the deformation of specialized materials (e.g., PVDF with BaTiO_3_ in [18]) for force measurements. In general, piezoelectric sensors do not measure static loads well [9], and in some instances, the choice of material can limit the bandwidth of the dynamic load measurement [18]. Both piezoelectric and triboelectric sensors, the latter of which generates electric fields as materials with differing electronegativities, move relative to one other [9,19] and exhibit self-powering benefits but require specialized materials and fabrication techniques for successful implementation [18,19]. Optical sensors are based on measuring wavelength changes of transmitted light caused by applied loads. A flexible optical sensor was demonstrated in [21], but they noted that its use in wearable applications is limited by the computational complexity required for accurate measurements. Finally, piezoresistive sensors measure forces through changes in material resistance under load [9,23]. These sensors can be customized for specific scenarios with simple fabrication, small size, and flexibility [9], and have the benefit of simple readout [9,23,25,26], high sensitivity [9,23] and low cost [7] but often provide less accurate measurements.

As a continuation of our telementoring work, we want to design a custom-built system that can monitor forces applied by the trainee with full hand measurements (including the palm, rather than just the fingertips), while containing the cost and complexity of both the fabrication process and the final system, which is a desirable quality in real-world medical applications [27,28]. Other important requirements are that the measurement system should have minimal impact on hand kinematics and sensation, which requires sensors with low thickness [6], and that it should be flexible enough to stabilize grip [8] and increase comfort while the system is worn [23]. At the same time, we want to obtain appropriate accuracy for the measurements obtained. These are conflicting requirements, which require a choice between trade-offs. In previous work, we used commercial FSR sensors [12] to measure forces, and found that while the accuracy was (mostly) sufficient for our requirements, their design and construction made them ill suited for use in a more complex setup with multiple sensors embedded in a glove. This led us to look into the design of custom force sensors, where, generally speaking, one of two possible directions could be taken: (i) design an expensive, potentially complex but high-end and accurate wearable sensing system, or (ii) design a customizable system that performs at least as well as FSR sensors but has a comparable or even lower cost and is as simple as possible to fabricate. The latter is our choice.

This choice makes Velostat (Desco Industries, Chino, California, USA), a piezoresistive composite material sold in fabric-like sheets, a particularly appealing material. Indeed, sensors fabricated from Velostat have garnered increased interest in recent years [3,7,13,29,30]. These sheets consist of a polyethylene film embedded with carbon powder, which moves within the polymer matrix under load, decreasing the resistance of the composite [22,25,26,29]. The potential of Velostat as a force sensitive material has been well documented, with applications ranging from measuring contact forces inside a prosthetic socket [25], grip [31] and environmental contact [32] forces for robots, to measuring the pressure on the bottom of feet [33,34], as well as many other applications [35,36,37,38]. The primary advantages of using Velostat in tactile sensors are its ability to be customized to the application, low cost, low profile (0.1 mm thick), and mechanical compliance and flexibility [29]. However, Velostat has been shown in previous work to have issues with performance, specifically when it comes to accuracy [22,25], drift [25,39], and repeatability [25], which is something that we experienced ourselves in our first attempts of using the material. In this paper, we focus on evaluating potential increases in accuracy from stacking multiple layers of Velostat, past the limited investigation of the effect of using two and three layers that was conducted in previous work [34], and specifically on identifying a specific target number of layers that can be used to design modular sensors for measurement of forces in hands, keeping in mind the trade-off between accuracy and use of low thickness of sensing material to preserve perceptual abilities of the user.

## 2. Motivation

As stated in previous section, our goal is to design sensors that satisfy the following requirements:

1. They should be low cost (with a per-sensor cost comparable to or less than that of a typical FSR commercial sensor);
2. They should offer measurements at least as accurate as those that can be obtained from FSR sensors;
3. They should be easily customized with minimal fabrication effort.

**Figure 1:**
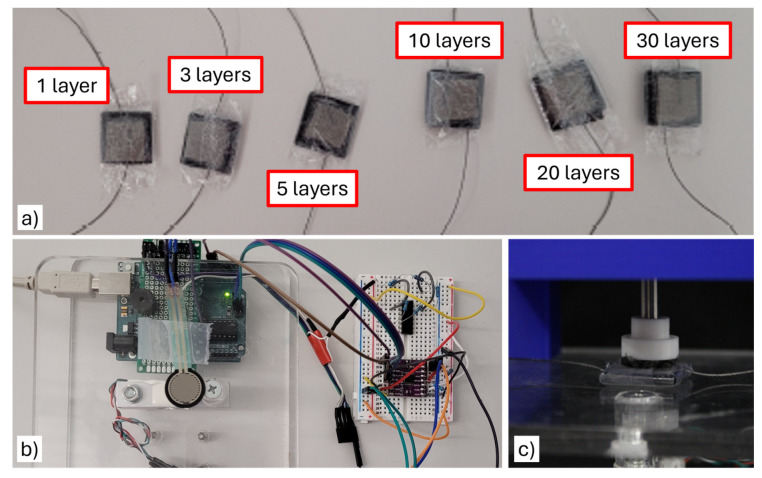
Overview of the force sensors evaluated in this paper. (**a**) Velostat sensors built with 1, 3, 5, 10, 20 and 30 layers of Velostat. (**b**) FSR 402 commercial sensor attached over the load cell used as ground truth. (**c**) Close-up view of the 20 layer sensor during one of the evaluations.

With these design requirements in mind, our first attempt was to use simple resistive sensors fabricated from a single layer of Velostat sandwiched between conductive fabric (shown in Figure 1 as the leftmost sensor). However, preliminary evaluation with this simple sensor indicated a worse performance in terms of accuracy when compared with the commercial FSR sensor used in [12]. However, we noticed that stacking layers of standard sheets of Velostat improved accuracy, which led us to the fundamental question for this paper: does stacking layers of Velostat improve measurement accuracy, and if so, is there a point of diminishing returns from adding layers?

It is generally known that increasing the thickness—and therefore the base resistance—of a sensor will lead to an increased change in resistance per unit force [38], improving measurement capabilities [9,22]. Multilayered designs for Velostat sensors have been explored in a limited capacity, with [34] reporting similar performance from two- and three-layer sensors built from a standard Velostat sheet (thickness 0.1 mm) but not considering designs with a number of layers greater than three. Our proposed approach expands on this work by considering the effect on the accuracy of additional standard 0.1 mm layers of Velostat.

In adding layers, a major goal is to keep the fabrication process simple as described above in the definition of our design requirements. The simplest way to fabricate a multilayered sensor is to stack layers with no additional material in between. However, a possible concern with that is that the layers could move with respect to each other, or might have non-ideal contact conditions. This was considered in [34], where the authors found that using conductive adhesive between layers increased the base resistance, making it easier to detect small variations in force, but also created repeatability issues as evidenced by a high variability in resistance that they observed for the same values of force. Adding additional layers of Velostat will also generate a higher base resistance, which compensates for the main drawback observed in [34] from not using a conductive adhesive. With this in mind, the decision to not use an adhesive is made in the interest of keeping cost low and fabrication simple, and to prevent potential repeatability issues, and we instead package the sensors with wrap as described more in details in the next section.

For what concerns the evaluation of our proposed design, part of the problem that we are trying to solve is the trade-off between improving the accuracy of measurements and increasing the thickness of the sensor. As tactile sensors for measuring forces on the hand should limit impairment of tactile sensing for the user, it is desirable to have a low thickness for the sensor [6], leading to our interest in identifying any point of diminishing returns for any potential improvement in accuracy from increasing the number of layers. To better place the performance of these sensors in context, we also provide quantitative comparisons between these multilayered sensors and a FSR 402 sensor (Interlink Electronics, Fremont, CA), where error for both is measured against a reliable baseline comparison provided by a commercial load cell. We consider a few different loading conditions: (i) a static evaluation, where masses are applied to the sensor in a randomized sequence; (ii) a cyclic evaluation, where an indentation device is used to apply a periodic force; and (iii) a finger-based evaluation, where a group of participants is asked to interact with the sensor by pressing against it with their fingers. Data are then analyzed and used for statistical analysis comparing differences in measured error (with respect to the load cell) for the Velostat sensors and the FSR sensor. This type of analysis was under-explored in previous work, where comparisons of this kind have been largely qualitative [34,38].

## 3. Hardware

### 3.1. Sensing Unit Construction

All Velostat sensors were fabricated with a layered design as shown in the graphical abstract for this paper, with conductive fabric layers on the two ends of the sensors and a variable number of standard 0.1 mm Velostat layers in the middle. Figure 1 shows an overview of the sensors that we consider. Sensors with 1-, 3-, 4-, 5-, 10-, 20-, and 30-layers are constructed to provide observations on the effect of the number of layers on sensing performance (Figure 1a). This range of number of layers is chosen to explore the trade-off between increased accuracy and thickness, where we expect to see diminishing returns at or before the 30-layer design. In addition to the custom Velostat sensors, an FSR 402 sensor from Interlink is included in the comparison (Figure 1b) to provide a term of comparison against a sensor of comparable low-cost design for force sensing in hands. A commercial load cell is used as a reference to compare against.

**Figure 2:**
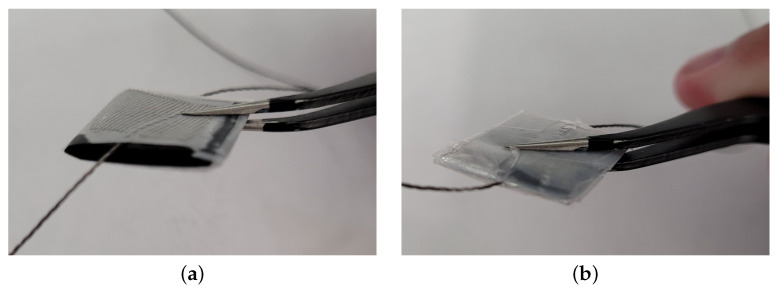
Force sensors fabrication. (**a**) Close-up view of the 4-layer sensor right with the layers assembled before the final assembly with cling wrap. (**b**) As a final step, the sensors are wrapped in cling wrap, which prevents the layers from moving with respect to each other.

The Velostat sensors are constructed as follows. We use a square, 18 × 18 mm scrap-booking hole punch to cut the material in squares, to have consistency in shape and size for each layer of Velostat. The silver-coated conductive fabric is used as an electrode and cut into 14 mm-wide squares in contact with the Velostat, with a length of 18 cm of silver conductive thread used for the leads for each sensor. The whole assembly is packed together with clear tape (Figure 1d), then wrapped in cling wrap to add an extra layer of protection (Figure 1e). No adhesives are used between layers in the interest of keeping fabrication simple and to prevent possible confounding effects from differences in the application—instead, the final assembly of the sensors is performed by wrapping them in cling wrap, which ensures that the layers stay in contact with each other during use (Figure 2).

In [34], which considered layered designs up to three layers, authors reported an increase in base resistance when using anisotropic conductive film as an adhesive between layers, accompanied by worse repeatability. We considered the use of an adhesive for our layered sensors, but we did not observe an improvement in performance in a pilot evaluation. This, combined with results from [34] and the fact that we wanted to keep cost low and fabrication simple, led us to prefer the simpler and more economic design approach using cling wrap to keep the layers together suggested in this work. In light of this evidence, while we acknowledge that a more in-depth evaluation would be required to conclusively show that use of an adhesive is not beneficial when compared to our unglued approach, we make the deliberate choice of focusing on sensors built using the simpler fabrication method in this work.

### 3.2. Sensor Reading Circuit

Sensor readout is achieved by measuring the voltage across the sensor in a simple voltage divider circuit connected to the Arduino. Because of the difference in thickness, each of the Velostat sensors has a different base resistance, generally requiring a different static resistor in the voltage divider. The resistors are chosen through a standard process that aims to maximize sensitivity for the target force range (0 to 10 N in this case [6,40,41]), which is performed as outlined in the FSR 402 integration guide [42] for all sensors. The chosen resistor values are shown in Table 1.

### 3.3. Load Cell and Loading Platform

**Figure 3:**
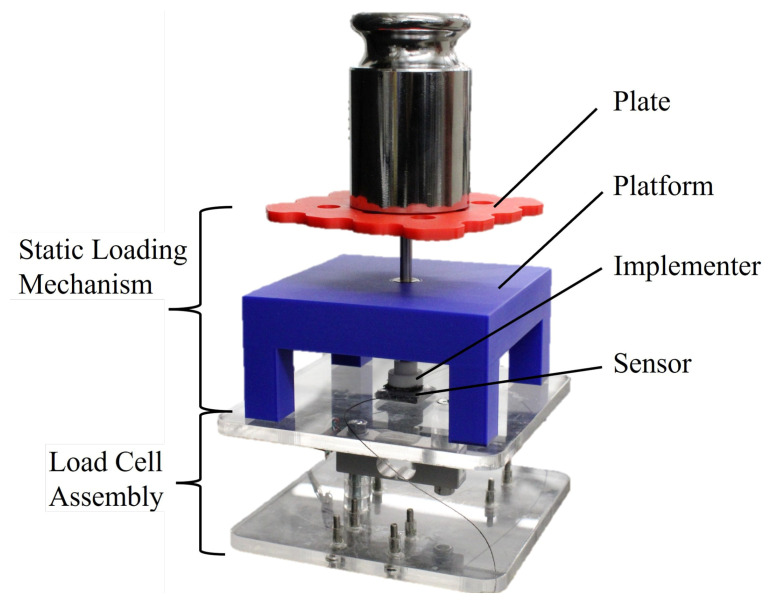
Load cell experimental setup for sensor characterization and weight-based evaluation.

Figure 3 shows the 5 kg load cell (Sparkfun Electronics, Niwot, Colorado, USA) that we use as ground truth, mounted between two acrylic plates and connected to an Arduino Uno through a 24-bit ADC module for reading differential voltages (CJMCU-1220 ADS1220 ADC I2C Low Power 24 Bit A/D Converter Module). It also shows the weight application mechanism, which is used to ensure that weights are applied to the sensitive area of each sensor. The load cell is calibrated linearly on data obtained by placing masses individually and directly on the acrylic surface for 20 s each. The resolution of this load cell setup is 0.01 N. The masses used come from a weight set, and are combined to increase sequentially from 10 g to 50 g, 100 g, then proceeding in 100 g increments up to 1 kg. This corresponds to forces up to approximately 10 N, which is consistent with typical forces on the hand as reported in the state of the art [3,6,12,31].

For calibration and the static evaluation, the loading mechanism shown in Figure 3 is used to ensure that the load is uniformly distributed across the sensing area for the Velostat sensors and the FSR 402. This mechanism consists of a 3D-printed loading plate, a four-legged platform, and an implementer which is used to enter contact with the sensor as shown in Figure 3. The loading plate and implementer are connected via a 5 mm stainless steel rod, with the motion restricted to be purely vertical by a linear bearing embedded in the platform. The portion of the implementer touching the sensor has foam fixed to it in order to lower the break/activation force described by the FSR manufacturer [42], with the added benefit of simulating contact against soft skin (e.g., a finger). The load cell measurements are bias-corrected for the weight from the four-legged platform (≈0.85 N) during calibration and static evaluation, as this force is not applied to the sensors being evaluated. However, when calibrating the sensors, it is essential to include the total weight from the steel rod, implementer with foam, and loading plate (≈0.37 N).

### 3.4. Continuous Load Application Mechanism

**Figure 4:**
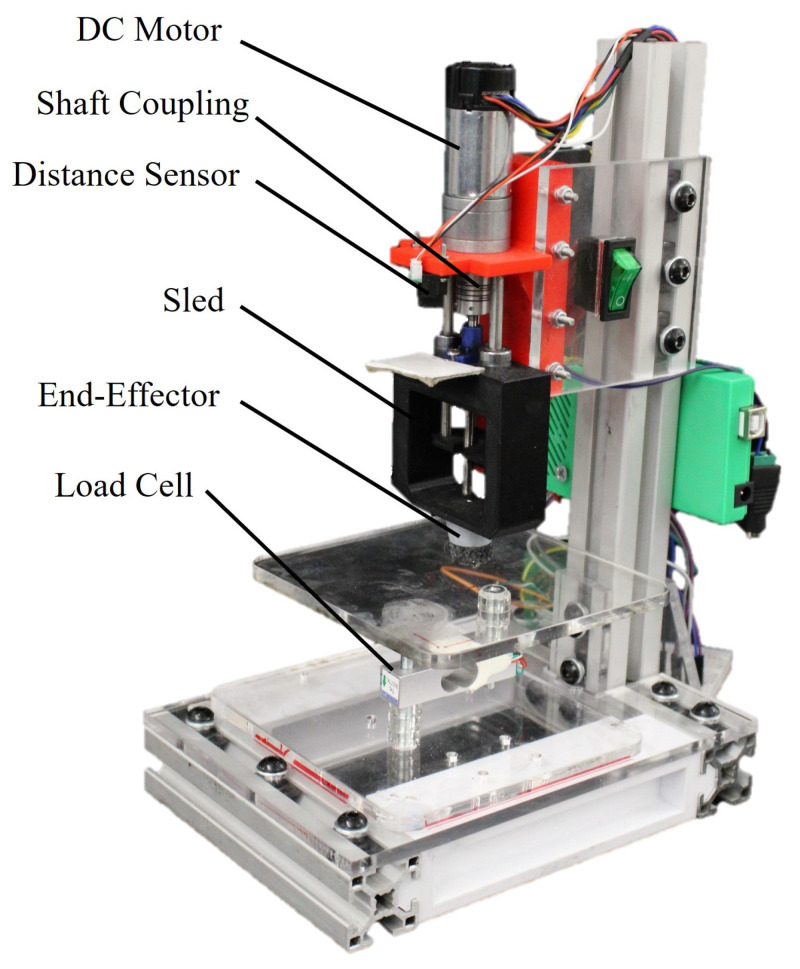
The automated loading mechanism used during the cyclic evaluations.

Figure 4 shows the mechanism that was custom built to apply continuous and consistent loading profiles in the cyclic load test. Forces are applied by a 6 V DC motor (Pololu, Las Vegas, Nevada, USA) turning an ultra-fine pitch lead screw (McMaster-Carr, Elmhurst, Illinois, USA), moving the end-effector, which has a contact interface similar to the one used in the static loading mechanism. The sled is connected to translate as the screw turns, with two 5 mm steel shafts and mounted bearings ensuring smooth vertical movement. A distance sensor is included to return to a consistent position at the end of each trial, while readings from the load cell are used to achieve closed-loop force control with an appropriately tuned PID controller.

### 3.5. Velostat Conditioning and Calibration

Sensors built with Velostat achieve better performance if they are mechanically conditioned prior to data capture [22,25]. The FSR 402 sensor similarly benefits from being conditioned [42]. This is accomplished by the experimenter for all sensors by alternating pressing and releasing a finger on the sensor in succession for around 10 to 20 s before any data acquisition. It is worth mentioning that, while conditioning could also be performed with a mechanical device like the indenting mechanism used in one of our evaluations, this would not represent a realistic procedure for actual use of the sensor in real-world settings. As mentioned later, we compensate for potential uncertainties, including those caused by manual conditioning, by having multiple data collection sessions for all our evaluations, allowing us to capture sources of variability in our statistical analysis.

To calibrate the sensor over the range of interest, the same masses used to calibrate the load cell (10 g, 50 g, then 100 g to 1 kg in 100 g increments, approximately covering the 0–10 N range of interest) are each applied for 10-second intervals, following a calibration procedure similar to those used in a previous work in the state of the art that used Velostat [33,35]. Loads are applied in a sequence of increasing magnitude from 10 g to 1 kg (loading) as well as in a sequence of decreasing magnitude from 1 kg down to 10 g (unloading). The calibration data are obtained from a loading–unloading sequence, followed by an unloading–loading sequence for all sensors. The masses are applied with the aid of the loading platform mechanism described earlier to ensure that all of the force applied is transmitted through the sensor.

There are a few different models for the relationships between the electrical properties of the material and the forces being applied that have been presented in the state of the art. While models that incorporate viscoelastic behavior are more accurate for higher force ranges [32,34,39], the current evidence from the state of the art suggests that a linear relationship between applied load and the conductance (equivalent to an inverse linear relationship between load and resistance) is appropriate for force ranges between 0 and 15 N [31,32,33,38,39]. Following these results, we map load cell readings to the corresponding sensor conductance with a linear fit in the calibration phase. For all calibration fits that are obtained and used as part of the work in this paper, the R-squared is 0.95 or higher.

## 4. Experimental Evaluation

In this section, we describe the experimental design that was used to evaluate the performance of the custom Velostat sensors fabricated with different numbers of layers, as well as the commercial FSR 402 sensor, using the hardware described in Section 3. Because our intended application is the measurement of forces in hands, we focus on force ranges (0 to 10 N) and loading rates (typically < 10 N/s) that are typical in grasp and manipulation tasks [6,40,41]. We consider three different evaluations: a static evaluation with masses, a cyclic evaluation with a periodic force applied by the indenter described in the previous section, and a finger-based evaluation obtained in a short user study where participants pressed on the sensor with their index finger. Throughout this section, we refer to the Velostat sensors by their number of layers followed by the letter L (so for example 10 L is the ten-layer Velostat sensor), while the FSR 402 sensor is labeled simply as FSR. Because we are aiming for a low-cost system, and trying to keep both fabrication and use procedures simple, and because Velostat is known to suffer from repeatability issues, we expect to see variability in the data. For this reason, all experiments presented in this section are conducted in different sessions on different days, allowing us to capture this variability for all sensors.

### 4.1. Static Loading Evaluation

The purpose of the static evaluation is to evaluate the accuracy for each sensor under static loading conditions, which are obtained by placing a set weight on each sensor for a certain amount of time. At the start of this evaluation, the sensor is first conditioned and calibrated. The same weight application mechanism used for the calibration (Figure 3) is kept on the sensor and the load cell, and the same 12 levels of weight used during the calibration (10 g, 50 g, then 100 g to 1 kg in 100 g increments, approximately covering the 0–10 N range of interest) are applied to each sensor in a randomized sequence. This is repeated ten times, using the same set of ten randomized sequences for each sensor. This choice is motivated by the fact that Velostat is known to experience drift under static loads [25,39], leading us to want to minimize the risk of the order of masses being applied being a confounding factor for differences between sensors.

**Figure 5:**
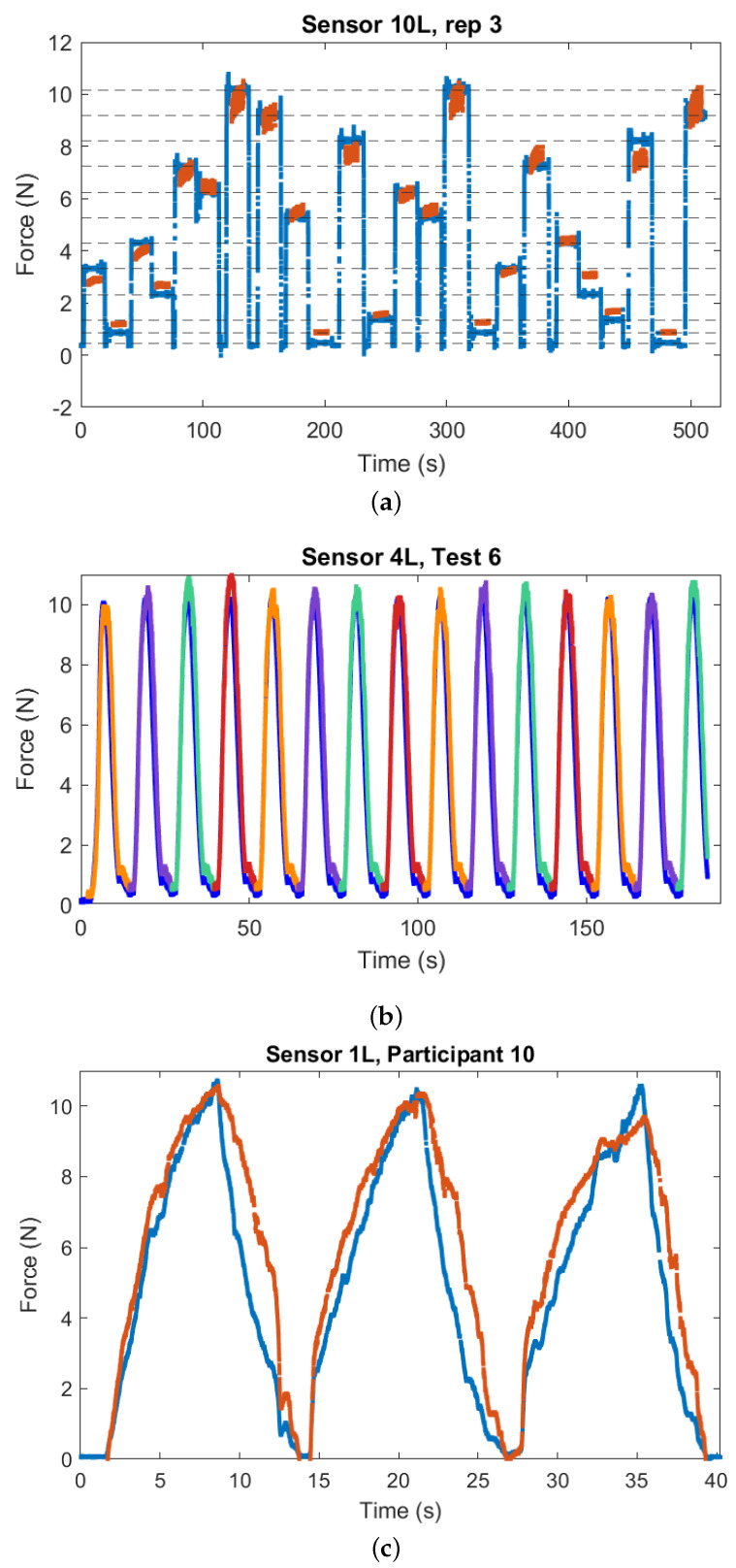
Representative plots from the experimental evaluation. (**a**) Representative trial from the static evaluation. Load cell readings in blue, segmented readings from the 10 layer velostat sensor in red. (**b**) Representative trial from the cyclic evaluation. Load cell readings in blue, readings from the 4 layer sensor segmented for each cycle are overlapped in multicolor. (**c**) Data from participant 13 interacting with the 1-layered Velostat sensor. The load cell reading is shown in blue, while the corresponding reading from the Velostat sensor is in red.

Each load is kept on the sensor for at least 10 seconds. For each weight application, the error is calculated as the root mean square error (RMSE) between steady-state force readings from the sensor being evaluated and readings from the load cell (yielding a total of 12×10=120 steady state RMSE measurements for each sensor). The steady-state force readings are obtained by segmenting the data based on force changes generated by the experimenter when removing and placing weights, and then selecting the central 10 s of data for each segment, corresponding to samples for which the mass is placed on the sensor in a steady-state condition. Figure 5a shows a sample plot from the third repetition on the 10-layer sensor.

### 4.2. Cyclic Evaluation

For this evaluation, we use the indenter shown in Figure 4 to apply a sinusoidal load with amplitude 10 N and period 10 s on each sensor, with 15 cycles applied per test. The use of the indenter mechanism tracking a specific force profile is intended to produce consistent loading profiles for each sensor across all iterations of this evaluation. Each sensor is calibrated and conditioned before each test. Six of these tests are performed for each sensor on two separate days (with three consecutive tests each day), and the RMSE between readings from the sensor and the load cell were calculated across each loading cycle, providing a total of 15×6=90 overall RMSE values across each cycle for each sensor. The segmentation for this experiment is obtained from the known force profile produced by the indenter. Figure 5b shows data from the sixth test ran on the four-layered Velostat sensor, with segmented data from the 4L sensor overlapped to the load cell reading.

### 4.3. Finger Evaluation

This evaluation is intended to evaluate the ability of the sensors to measure forces in a scenario closer to the intended real-world application, i.e., force interactions from a human hand. For this purpose, we recruited 12 healthy participants, (3 female, age 25±2.84, 1 left-handed). The study was approved by the Institutional Review Board of the University of Utah under IRB number 00154923. The sensors are calibrated and conditioned before each session as in the previous evaluations. During the experiment, participants are shown a real-time readout of the force that they are applying on screen, and are told to press against the sensor with the index finger of their dominant hand until they reach 10 N, then raise the finger back, decreasing the force to zero. Participants are instructed to interact naturally with the sensor, without sticking to a fixed speed but to not release the finger instantly from the sensor so that we can also capture data for the unloading part of the cycle. Participants are also instructed on how to press on the Velostat sensing surface (e.g., to not press on the edges, as this would not measure part of the force being applied), and to complete three loading/unloading cycles for each sensor. The choice to limit constraints on participant movements is motivated by the fact that we already have data on a dynamic but consistent load from the cyclic evaluation, and the goal for this final evaluation is to obtain data that are more representative of a real-world use case for our proposed system.

## 5. Results

**Figure 6:**
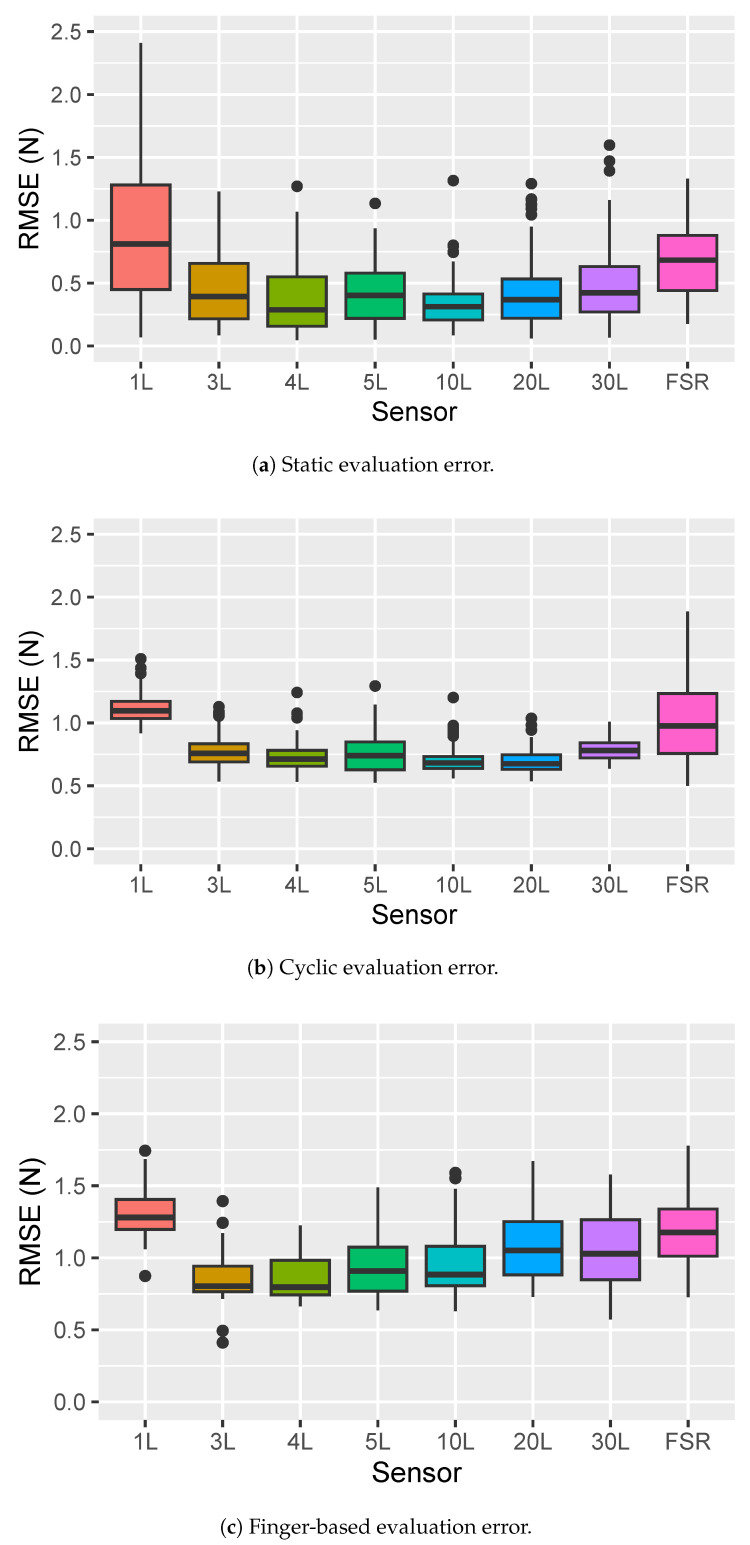
Box plots showing the effect of the type of sensor on reading errors with respect to the load cell. Velostat sensors are labeled as the number of layers used in their construction followed by the letter L. The FSR 402 commercial sensor is labeled as FSR.

Figure 6 shows box plots for the results of each evaluation. The box plot for the static evaluation (Figure 6a) shows strong indication of inhomogeneous variances between each sensor (in particular, the 1 L sensor seems to have a much higher variability). The 1 L and FSR sensors have the highest errors on average, while the 4 L and 10 L sensors seem to performed best. We do observe, as expected from previous results in the state of the art [25,39], some indication of drift from the static load (see Figure 5a as an example).

Figure 6b is a box plot for the cyclic evaluation, for which we observe in general lower variability in the data (perhaps motivated by the fact that the velostat sensors are not as susceptible by drift), although the box plot for the FSR error shows a much higher level of variability. The multilayer velostat sensors appears to perform best and most consistently in this evaluation, with the lowest average RMSE being measured for the 10 L and 20 L designs.

Finally, a box plot for the RMSE values for the finger-based evaluation is shown in Figure 6c. Differences in variability across sensors are less noticeable in this case, but the 1 L and FSR sensors still show the highest error. The 3 L and 4 L sensors show the lowest error in this evaluation.

Because of the potential for heteroskedasticity in some if not all of the comparisons, we decide to use Welch’s ANOVA to compare RMSE values across sensors for each of the tests. Welch’s ANOVA uses an adjusted F ratio that makes it robust to the dishomogeneity of variance. In addition, it has been shown to also be robust to violations of normality assumptions [43] but does not inflate the type I error rate like non-parametric tests (e.g., Kruskal–Wallis) tend to do when sample sizes within groups are equal [44]. For all assessments, it is found that the sensor being used has a significant effect on the RMSE (F(7,405.82)=24.23, $p\leq 2.2\times 10^{-16}$ for the weight-based assessment, F(7,303.74)=131.38, $p\leq 2.2\times 10^{-16}$ for the cyclic evaluation, and F(7,104.7)=18.27, $p=1.08\times 10^{15}$ for the finger-based evaluation—note that Welch’s ANOVA can yield decimal numbers for the denoninator degrees of freedom because of its correction factor).

To evaluate differences between levels, a post hoc comparison is performed with paired t-tests with non-pooled standard deviations and no assumption of equal variances, using Holm corrections for the *p*-value to account for inflated Type I error rates. The results of this post hoc analysis are shown for each evaluation in Table 2, where we order the factors decreasingly based on the mean RMSE observed. To make the results easier to interpret, we use a compact letter display (CLD) representation [45], where different letters are used to label the sensors based on the significance of post hoc comparisons of means. Levels labeled with multiple letters show no significant difference with respect to other levels with shared letters. For example, in Table 2a, sensor 4 L (labeled *de*) does not differ significantly from the sensors 3 L, 20 L, and 5 L (labeled *cd*) or 10 L (labeled *e*) but is significantly different from sensor 30 L (labeled *c*).

Overall, the results of the post hoc analysis confirm the qualitative analysis from inspection of the box plots, with similar general trends observed in all three evaluations. Specifically, the 1 L and FSR sensors show an indication of higher errors being associated to these sensors when compared to the others. Differences between the multilayered velostat sensors (3 L to 30 L) are less evident, but the 4 L and 10 L sensors seem to show the most consistent improvement in error across the different experiments, being the only two sensors that consistently show as part of the group of levels associated to the lowest error.

## 6. Discussion and Conclusions

In this paper, we have evaluated the effect of stacking layers of Velostat to build force sensors, with a focus on applications in measurement of forces for hands (forces up to 10 N), and the goal to achieve accuracy at least comparable to the one observed in FSR sensors for a smaller cost and with the ability to design custom sensors. We considered seven different designs built with Velostat and conductive silver fabric, each with a different number of layers of Velostat (1, 3, 4, 5, 10, 20 and 30 layers, cost for each sensor < USD 1). Additionally, we included the FSR 402 sensor in the comparison, a widely used commercial sensor designed for the measurement of forces in hands, which has a comparatively low cost (≈USD 10). Three different evaluations were carried out on the eight sensors: (i) a static evaluation, where randomized sequences of constant weights were placed on each sensor; (ii) a cyclic evaluation, where a periodic loading profile was applied by an indentation mechanism on each sensor; and (iii) a finger-based evaluation, where a user study with 12 participant was performed for each sensor, during which users pressed on them with their fingers with no constraints on the rate of force being applied. The evaluations included tests performed in different sessions and across different days.

Results from the three evaluations that we considered show consistent evidence of lower RMSE for the layered sensors, with the 1 L sensor always showing significantly higher RMSE (on average 0.89, 1.11, and 1.32 N for the static, cyclic, and finger-based evaluation, respectively), followed closely by the FSR (average RMSE 0.66, 1.03, and 1.17 N, respectively). All the layered designs consistently showed a statistically significant lower RMSE when compared to the FSR sensor and the 1 L design. While the differences between layered designs were less marked and not fully consistent across tests, we did observe that the 4 L and 10 L designs always performed at the same level across all tests, and outperformed the other designs in most cases (showing RMSE means of 0.38, 0.73 and 0.86 N for the 4 L, 0.33, 0.71 and 0.96 for the 10 L on the static, cyclic, and finger evaluations, respectively), as well as a reduction in error between 27% and 60% compared to the 1 L design.

Our results suggest that it is possible to reduce error in force sensors built with Velostat in a very simple but effective way by stacking multiple layers of the material. It is worth noting that in this paper, we used a very simple fabrication process, cutting the layers to the same size using a square hole punch implement, and then stacking them between two silver-coated fabric layers and wrapping them with tape and cling wrap. This process is very straightforward and could be easily repeated with no need of particular tools or fabrication equipment. As mentioned previously in [34], the authors observed that using conductive adhesive can increase base resistance but seems to worsen repeatability for a design using two or three layers. Further investigation is required to conclusively evaluate is this would also be true for the multilayered designs shown in our work—however, our results show that all our unglued multilayered designs outperform both the 1 L design and the commercial FSR 402 sensor.

There are some limitations that need to be kept in mind when considering the implications of this work. The first one is that we focused on force ranges and loading rates that are typically applied by fingers during manipulation (0 to 10 N, and typically <10 N/s [6,40,41]), meaning that the results we found in this work might not extend to interactions outside of these ranges. Additionally, the Velostat sensors built for this work were cut in 18×18 mm squares, a size that is appropriate for measuring forces at the fingertips or for localized contact information in other spots on hands. Larger or smaller patches might exhibit different behavior. Finally, for applications where the sensors are in contact with the human body, one might observe an effect on accuracy from heat exchange with the user. While the finger-based evaluation is able to incorporate potential effects from it, there was no contact with a user in the static and cyclic evaluation.

Within the context of these limitations, our work provides evidence that simply layering multiple Velostat sheets in the fabrication of sensors can reduce errors and improve the accuracy of data collected. This paves the path towards the development of low-cost distributed sensing that does not require complex fabrication or particular tools, and in addition to our intended application to telementoring could provide a powerful and approachable sensing solution for open source projects, community outreach activities, and other applications where cost is a concern and simple fabrication processes are desirable. Future work will focus on using Velostat in a custom sensing setup, specifically to measure forces from medical trainees as they are working on training scenarios in the context of remote telementoring. We will focus on using sensors built with four layers of Velostat since the 4 L design is one of the best performing, and having a lower thickness is desirable in sensing for manipulation. Additionally, we will further investigate other metrics of performance such as hystherisis, sensitivity, and drift with a focus on the 4 L sensor.

## Figures and Tables

**Table 1:** Resistors in the voltage divider for each sensor.

Sensor	1 L	3 L, 4 L, 5 L	10 L	20 L, 30 L	FSR
Bias Resistor	30 Ω	100 Ω	1 kΩ	3 kΩ	10 kΩ

**Table 2:** Post hoc pairwise comparisons of mean values of RMSE for each sensor, including adjusted *p*-values, compact letter display representation of differences and means for each level. Sensors are ordered decreasingly based on average RMSE. Values with $p_{adj}<0.05$ in bold.

(a) Results for the Static Evaluation.							
Mean RMSE (N)							
a	b	c	c d			d e	e
0.89	0.66	0.50	0.46	0.43	0.41	0.38	0.33
1 L	FSR	30 L	3 L	20 L	5 L	4 L	10 L
1 L	**0.00077**	$8.0\times 10^{-10}$	$7.8\times 10^{-12}$	$1.4\times 10^{-13}$	$2.9\times 10^{-15}$	$6.6\times 10^{-16}$	$<2\times 10^{-16}$
FSR	-	**0.00073**	$4.1\times 10^{-6}$	$2.3\times 10^{-08}$	$2.5\times 10^{-11}$	$1.8\times 10^{-11}$	$<2\times 10^{-16}$
30 L	-	-	1.00	0.446	0.0765	**0.0199**	$8.7\times 10^{6}$
3 L	-	-	-	1.00	0.700	0.226	**0.00053**
20 L	-	-	-	-	1.00	0.887	**0.0188**
5 L	-	-	-	-	-	1.00	**0.0378**
4 L	-	-	-	-	-	-	0.700
	$p_{adj}$ (Holm correction)						
**(b) Results for the Cyclic Evaluation.**							
**Mean RMSE (N)**							
**a**		**b**		**b c**		**c**	
1.11	1.03	0.79	0.78	0.75	0.73	0.71	0.70
1 L	FSR	30 L	3 L	5 L	4 L	10 L	20 L
1 L	0.1380	$<2\times 10^{-16}$	$<2\times 10^{-16}$	$<2\times 10^{-16}$	$<2\times 10^{-16}$	$<2\times 10^{-16}$	$<2\times 10^{-16}$
FSR	-	$9.5\times 10^{-9}$	$7.2\times 10^{-9}$	$8.1\times 10^{-10}$	$1.5\times 10^{-11}$	$3.9\times 10^{-13}$	$2.9\times 10^{-14}$
30 L	-	-	1.00	0.61	0.013	$1.4\times 10^{-5}$	$4.9\times 10^{-9}$
3 L	-	-	-	1.00	0.138	0.0019	$1.4\times 10^{-5}$
5 L	-	-	-	-	1.00	0.189	**0.021**
4 L	-	-	-	-	-	0.814	0.138
10 L	-	-	-	-	-	-	1.00
	$p_{adj}$ (Holm correction)						
**(c) Results for the Finger Evaluation.**							
**Mean RMSE (N)**							
**a**	**ab**	**bc**		**c d**		**d**	
1.32	1.17	1.08	1.07	0.96	0.94	0.87	0.86
1 L	FSR	20 L	30 L	10 L	5 L	3 L	4 L
1 L	0.322	**0.0051**	**0.0024**	$2.8\times 10^{-6}$	$6.8\times 10^{-8}$	$4.9\times 10^{-11}$	$4.5\times 10^{-12}$
FSR	-	0.796	0.796	**0.0346**	**0.0077**	$8.6\times 10^{-5}$	$3.2\times 10^{-5}$
20 L	-	-	1.00	0.61	0.286	**0.0055**	**0.0024**
30 L	-	-	-	0.751	0.360	**0.0077**	**0.0032**
10 L	-	-	-	-	1.00	0.751	0.587
5 L	-	-	-	-	-	0.796	0.751
3 L	-	-	-	-	-	-	1.00
	$p_{adj}$ (Holm correction)						

## Data Availability

Data available from the corresponding author upon request.
